# Cerebral Microbleeds Temporarily Become Less Visible or Invisible in Acute Susceptibility Weighted Magnetic Resonance Imaging: A Rat Study

**DOI:** 10.1089/neu.2018.6004

**Published:** 2019-05-06

**Authors:** Arnold Tóth, Zoltán Berente, Péter Bogner, Bálint Környei, Bendegúz Balogh, Endre Czeiter, Krisztina Amrein, Tamás Dóczi, András Büki, Attila Schwarcz

**Affiliations:** ^1^ Department of Neurosurgery, Pécs Medical School, Pécs, Hungary.; ^2^ Department of Radiology, Pécs Medical School, Pécs, Hungary.; ^3^ MTA-PTE Clinical Neuroscience MR Research Group, Pécs, Hungary.; ^4^ Department of Biochemistry and Medical Chemistry, Pécs Medical School, Pécs, Hungary.; ^5^ János Szentágothai Research Centre, University of Pécs, Pécs, Hungary.; ^6^ Research Group for Experimental Diagnostic Imaging, Pécs Medical School, Pécs, Hungary.; ^7^ Diagnostic Center of Pécs, Pécs, Hungary.

**Keywords:** brain trauma, microbleed, MRI, rat, susceptibility weighted imaging (SWI)

## Abstract

Previously, we reported human traumatic brain injury cases demonstrating acute to subacute microbleed appearance changes in susceptibility-weighted imaging (SWI—magnetic resonance imaging [MRI]). This study aims to confirm and characterize such temporal microbleed appearance alterations in an experimental model. To elicit microbleed formation, brains of male Sprague Dawley rats were pierced in a depth of 4 mm, in a parasagittal position bilaterally using 159 μm and 474 μm needles, without the injection of autologous blood or any agent. Rats underwent 4.7 T MRI immediately, then at multiple time points until 125 h. Volumes of hypointensities consistent with microbleeds in SWI were measured using an intensity threshold-based approach. Microbleed volumes across time points were compared using repeated measures analysis of variance. Microbleeds were assessed by Prussian blue histology at different time points. Hypointensity volumes referring to microbleeds were significantly decreased (corrected *p* < 0.05) at 24 h compared with the immediate or the 125 h time points. By visual inspection, microbleeds were similarly detectable at the immediate and 125 h imaging but were decreased in extent or completely absent at 24 h or 48 h. Histology confirmed the presence of microbleeds at all time points and in all animals. This study confirmed a general temporary reduction in visibility of microbleeds in the acute phase in SWI. Such short-term appearance dynamics of microbleeds should be considered when using SWI as a diagnostic tool for microbleeds in traumatic brain injury and various diseases.

## Introduction

The term cerebral microbleed (or microhemorrhage) refers to small extravascular collections of blood or blood products in the brain that are visible as signal voids in magnetic susceptibility sensitive magnetic resonance imaging (MRI) techniques.^1,2^ Such techniques include T2* gradient echo (T2* GRE) and most sensitively susceptibility-weighted imaging (SWI).^3^ Since the improvements of these tools, microbleeds have been recognized increasingly in various cerebral diseases.

In acute traumatic brain injury, microbleed detection may aid the diagnosis, severity, and prognosis assessment of hemorrhagic diffuse axonal injury.^4–12^ Unfortunately, results are heterogeneous, which detains clinical feasibility.^13,14^ A potential confounding factor may be the inconsistent MRI appearance of microbleeds over time, as a result of the different possible biophysical states of blood.

Such temporal MRI features of large volume bleedings (i.e., hematomas) have been characterized widely based on both human investigations and experimental studies, encompassing hyperacute to chronic stages.^15–23^ The T1, T2, and magnetic susceptibility properties are affected substantially by the form of hemoglobin, the presence of hemoglobin breakdown products, red blood cell membrane integrity, or clot formation.^15^ Because peripheral and central parts of hematomas are prone to different biochemical environments, at certain stages, these hemorrhage compartments show marked MRI signal differences.^15,19,24–26^

Based on these studies, however, microbleed MRI dynamics cannot be understood fully: the size of a microbleed may be indeed in a microscopic range^2,27,28^; consequently, the relative surface (surface area to volume ratio) of a microbleed is vast compared with macroscopic hemorrhages. Therefore, both the active and passive biochemical alterations of blood components in a microbleed may differ significantly.

Observations on possible short-term temporal MRI appearance changes of microbleeds are yet scarce.^29^ Previously, we reported human traumatic brain injury case series demonstrating acute to subacute expansion of traumatic microbleeds in SWI.^30^ Other case reports presented that traumatic microbleeds in MRI may become reduced in number or volume,^5^ disappear,^31^ or transiently disappear^32^ early after injury. In a study focusing on cerebral blood flow changes in an experimental rat closed head injury model, authors ancillary reported some cases when hypointense foci congruent with microbleeds disappeared and later reappeared.^33^

Based on these case reports, it is uncertain whether acute microbleed appearance changes are because of changes in blood presence (full degradation for disappearance, re-bleeding/progression for re-appearance and expansion), or because of change in MR visibility over time. Therefore, we aimed to develop an experimental microbleed model suitable for the confirmation and characterization of short-term microbleed MRI appearance alterations. Histological processing was also performed to examine microbleed presence.

## Methods

### Rat microbleed model

Nineteen male Sprague Dawley rats weighing 350 g–450 g (Charles River Laboratories, Wilmington, MA) were used. The animals were maintained on a standard rodent diet with free access to water. All animals were previously healthy. All of the experiments and the general handling of the animals were approved by the National (Hungarian) Scientific Ethical Committee on Animal Experimentation (Number of permission: BA02/2000-69/2017 - valid for five years). All the procedures fully complied with national and international standards especially with Decree No. 40/2013 (II. 14.) of the Hungarian Government and EU Directive 2010/63/EU on the protection of animals used for scientific purposes.

Anesthesia was induced in a bell jar for 5 min under 4% isoflurane (Forane, Abbott, Abbott Park, IL) and a 7:3 mixture of N_2_O/O_2_, and maintained using isoflurane decreased to 1–3% delivered through a nasal mask. Rectal temperature was measured with a rectal probe (RET-4, Physitemp Instruments, Clifton, NJ) and thermometer (BAT-12, Physitemp Instruments, Clifton, NJ). The animal temperature was held at 36–38°C using a silicon tube, shaped to form a cylinder around the animal, connected to a hot/cold water circulator device (Scanvac heat safe SHC 2000, Labogene, Lynge, Denmark).

The rat was placed in a laboratory standard stereotactic frame (Stoelting Co., Wood Dale, IL). A midline scalp incision was made, and the skull was exposed with blunt dissection. A burr hole was placed 2 mm lateral to midline, 5 mm posterior to bregma, on both sides. To elicit microbleed formation, the brain was pierced vertically in a depth of 4 mm from the dura, using a stainless steel needle (Hamilton, Reno, NV) with a diameter of 159 μm (34 G) on the right side and 474 μm (26s G) on the left side. No blood or material was injected. The burr hole was sealed with bone wax, the wound was closed, and the rat was immediately transferred to the MR scanner. The entire surgical procedure took approximately 30 min.

### MRI

Animals were anesthetized during MRI as well, with 1–3% isoflurane and a 7:3 mixture of N_2_O/O_2_ administered through a nose cone animal holder. The animal temperature was held at 36–38°C degrees using a digital circulator device (Thermo Scientific Haake SC100, Waltham, MA). Imaging was performed on a Bruker (Bruker) 4.7 T Biospec 47/16 scanner with a receive-only 2 × 2 surface array coil for rat brain (Bruker). After obtaining a three-plane gross scout imaging, high resolution T2 weighted imaging was performed also in three planes to localize injury sites precisely (RARE sequence repetition time/echo time [TR/TE] = 2428/ 36 msec, RARE factor = 8, echo spacing = 12 msec, number of averages = 2, image matrix = 256 × 256 FOV = 35 × 35 mm^2^). Previous to acquiring SWI, map shimming was performed covering the brain with an ellipsoid volume. The SWI slice volume was adjusted centered to lesion sites based on the T2 images. The SWI was performed using the following parameters: three-dimensional (3D) acquisition type FcFLASH sequence, TR/TE = 29/11 msec, number of averages = 2, image matrix = 192 × 192 × 48, FOV = 32 × 32 × 8 mm^3^, flip angle = 12 degrees, mask weighting = 4 with gauss broad = 0.33 mm). Filtered phase data were stored.

As a pilot, three animals underwent imaging at five time points (immediate, 12 h, 24 h, 48 h, 125 h after injury). Based on the findings of these images and adhering to scanner availability, three imaging time points were set as immediate, 24 h, and 125 h for the further 16 animals. Of these 16 animals, 10 completed the three time points and were euthanized under anesthesia for histopathological evaluation. Three animals were euthanized after the immediate, and another three after the 24 h imaging.

### MRI data analysis

To visualize hypointense lesions without partial volume effects or dependence on slice orientation, ParaVision Acquisition 6.0.1 software (Bruker) was used to create coronal plane minimum intensity projection (minIP) images of 0.2 mm thick sections of the 3D SWI volumes. These sections were placed on the site of injury based on the signs of the skull burr holes and anatomy. The sections were checked to include all hypointensities related to the injury site. The minIP images were exported upsampled to a matrix of 768 × 768. To visualize 3D filtered phase data, the same steps were performed except using maximal instead of minimal intensity projection.

**FIG. 1.**
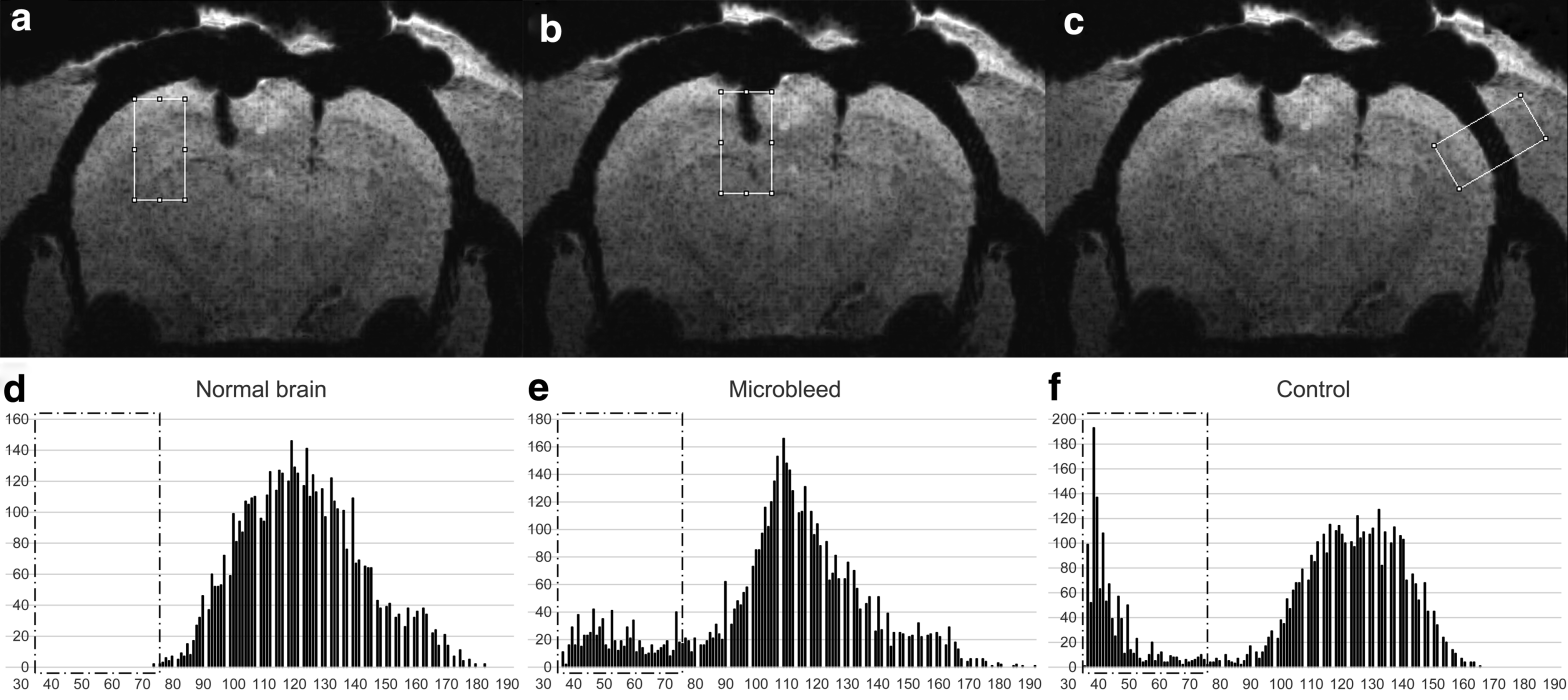
Illustration of the applied regions of interests (ROIs, overlaid on an immediate time point minimal intensity projection image) and their histograms with dash-dot lined boxes indicating “hypointense voxel” range. (**a, d**) Normal brain ROI and histogram, used to define hypointensity: the average value of the lowest intensities over normal brain (including all animals' immediate time point images, not only the presented one) was found to be 76; therefore, voxels with an intensity of ≤75 were defined as hypointense. (**b, e**) Microbleed ROI and histogram, used to calculate the number of hypointense voxels at injury sites corresponding to microbleed volumes (applied at all time points). The presented microbleed ROI included 791 hypointense voxels (see box in histogram). (**c, f**) Control ROI and histogram, for calculating the hypointense voxel number (applied at all time points) of the hypointense structure parietal bone assumed not to undergo volume changes over time. The presented control ROI included 1,202 such hypointense voxels (see box in histogram).

Quantitative analysis of hypointense lesion extent referring to microbleeds was performed using ImageJ software version 1.51k (Wayne Rasband, National Institutes of Health).^34^ A stack was built from all animals all time point SWI minIP images. First, voxel hypointensity was defined: in immediate time point minIP images, a rectangle shaped region of interest (ROI) of 50 voxels width ( = 2.1 mm) and 100 voxels height ( = 4.2 mm) was drawn and fitted over the normal appearing brain parenchyma (next to but excluding lesion sites, also excluding large vessels) in all animals (Fig. 1a). Using such ROIs, histogram analyses were applied to find the lowest included intensities (Fig. 1d). The average value of these lowest intensities was found to be 76; therefore, voxels with an intensity of ≤75 were defined as hypointense.

This ROI at identical sites and histogram analysis were also applied to the 24 h and 125 h minIP images to calculate average intensities. Based on these intensities, 24 h and 125 h image intensities were normalized to the immediate image intensity for each animal. Then, another rectangle shaped ROI of 50 voxels width ( = 0.2 mm) and 100 voxels height ( = 0.42 mm) was created. This ROI was placed over the injury sites (Fig. 1b), based on burr holes if the microbleed not visible, blinded to imaging time points, to extract the number of hypointense (≤ 5) voxels referring to microbleeds (Fig. 1e). Ventricular hemorrhage was included, if present.

Bone was used as a control structure, because bone appears hypointense in SWI but can be assumed to not undergo any volume changes over the 125 h investigation period. Therefore, a control ROI with identical dimensions as lesion ROI was placed over the lateral part of the parietal bone at the same level as the injury site and was rotated perpendicular to the bone (Fig. 1c). The number of hypointense voxels of the bone under the control ROI was extracted using the same intensity threshold value of 75 (Fig. 1f).

The number of hypointense voxels over both side lesion sites (i.e., microbleed extents) and parietal bone (control) across the immediate, 24 h, and 125 h time points were compared using repeated measures analysis of variance (ANOVA) with Bonferroni correction using Medcalc ver. 13.0.0.0 (MedCalc Software bvba, Ostend, Belgium).^35^ Such corrected *p* value less than 0.05 was considered statistically significant. Thirteen animals were available with the necessary three imaging time points for this analysis (the other six animals of the overall 19 animals were euthanized at earlier time points).

### Histology

After euthanasia, transcardial perfusion was performed with isotonic saline followed by 4% paraformaldehyde. That was performed in three cases after the immediate MRI acquisition, in another three cases after the 24 h acquisition, and after the 125 h imaging time point for the rest of the animals. The formalin fixation of the heads was continued for at least two weeks. Then, the brains were removed from the skull and were embedded in paraffin. If lesion sites were visible on the surface of the brain, the surrounding region was cut into 6 μm thick coronal sections and stained using Prussian blue. When lesion sites were not visible, the coronal sections were cut from the region supposed to contain lesions based on anatomy, and only every 10th section was first stained with Prussian blue to identify sections including the bleedings in the largest volume. Second, the adjacent sections were also stained to capture the entire bleeding. Bleedings were defined as extravasated red blood cells, or hemosiderin reaction at the lesion sites.

## Results

**FIG. 2.**
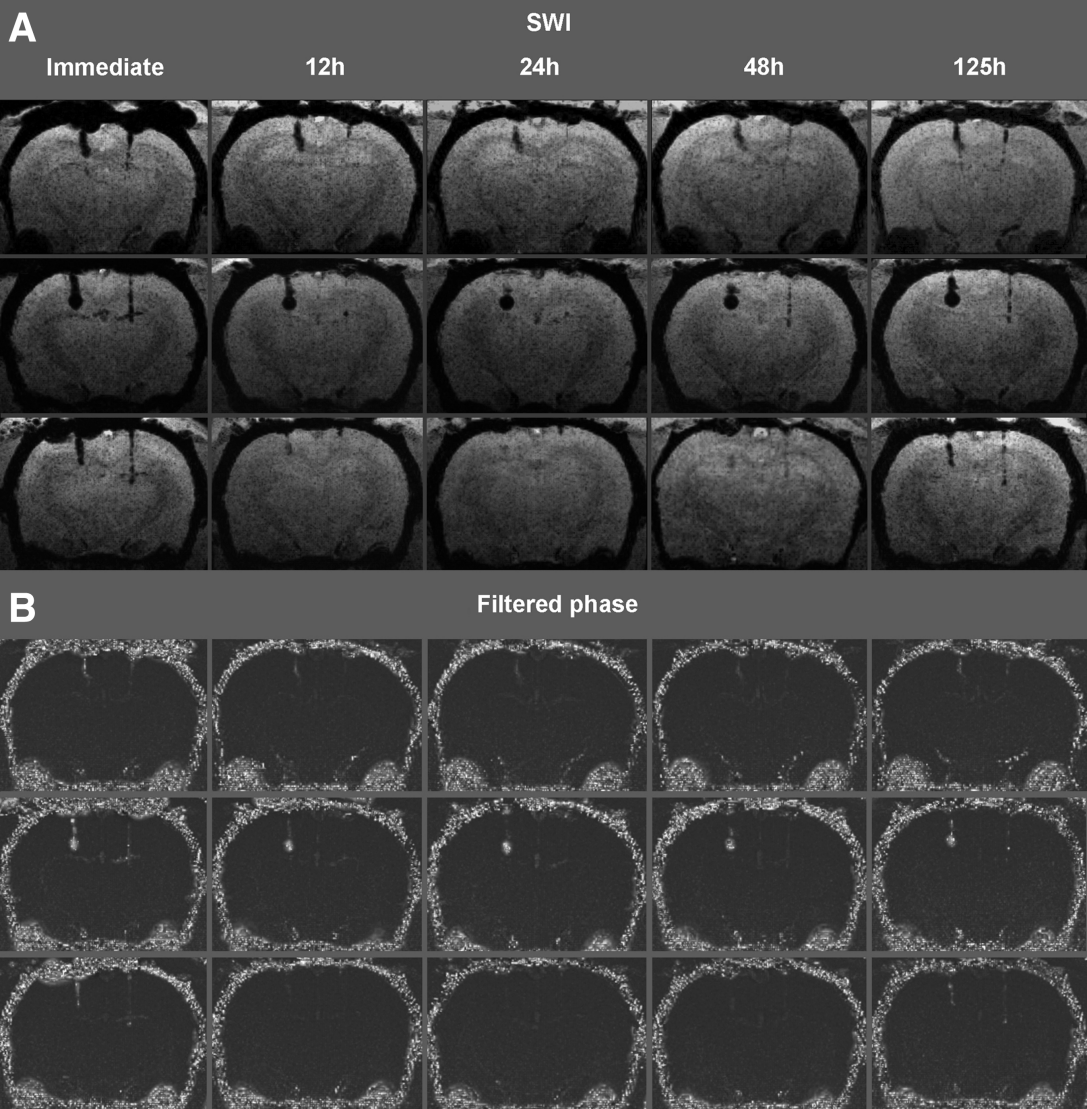
(**A**) Coronal susceptibility-weighted imaging (SWI) minimum intensity projection images of three rats that underwent five imaging time points (each row represents different animals). Hypointensities along both the left and right side brain parenchymal injury sites were present in the immediate acquisition. The hypointensities became smaller or completely absent in the 12 h, 24 h, and 48 h acquisitions. In the 125 h acquisitions, the hypointensities were again present in a similar shape, extent, and intensity as at the immediate time point. In the second animal, at the larger gauge injury track (left), a relatively large circumscribed blood collection was formed showing no noticeable change over time. (**B**) Coronal maximum intensity projection images of the same rats' filtered phase images. Hyperintensities consistent with phase alteration at lesion sites are more pronounced at the immediate and 125 h imaging time point than at the 12 h, 24 h, or 48 h time points.

**FIG. 3.**
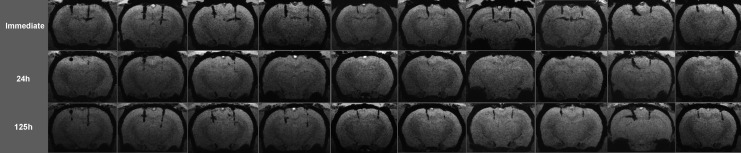
Coronal susceptibility-weighted imaging minimum intensity projection images of 10 rats that underwent three imaging time points (each column represents different animals). Hypointensities along both the left and right side brain parenchymal injury sites were present in the immediate acquisition. The hypointensities became smaller or completely absent at the 24 h time point and were again present at the 125 h time point, with a similar shape, extent, and intensity as at the immediate time point. In the first and ninth animals, at the larger gauge injury track (left), relatively large circumscribed blood collections were formed showing no noticeable change over time.

Hypointensities referring to microbleeds along both the left and right brain parenchymal injury sites were present in the immediate minIP images of all animals (Fig. 2A, 3). According to the lesser (right side) and greater (left side) gauge injury, the right side hypointensities appeared generally smaller than the left side ones. Occasionally, small amounts of blood entering the ventricles were observed. In the first set of animals that underwent five imaging time points, the hypointensities became smaller or completely absent in the 12 h, 24 h, and 48 h acquisitions (Fig. 2A). In the 125 h acquisitions, the hypointensities were again present of similar shape, extent, and intensity as in the immediate time point. The further 10 animals that underwent three time point imaging presented the same temporal characteristics: Hypointensities were present in the immediate imaging, decreased in extent or completely disappeared at the 24 h time point, and appeared the same as in the immediate imaging at the last (125 h) acquisition (Fig. 3).

Complete lesion disappearance occurred in nine of 13 animals at the lesser gauge injury site, and in five of 13 animals at the larger gauge injury site. In three animals (second in Fig. 2A, first and ninth in Fig. 3) at the larger gauge injury track, relatively large hypointensities referring to relatively large (macroscopic) circumscribed blood collections were formed showing no noticeable change over time.

**FIG. 4.**
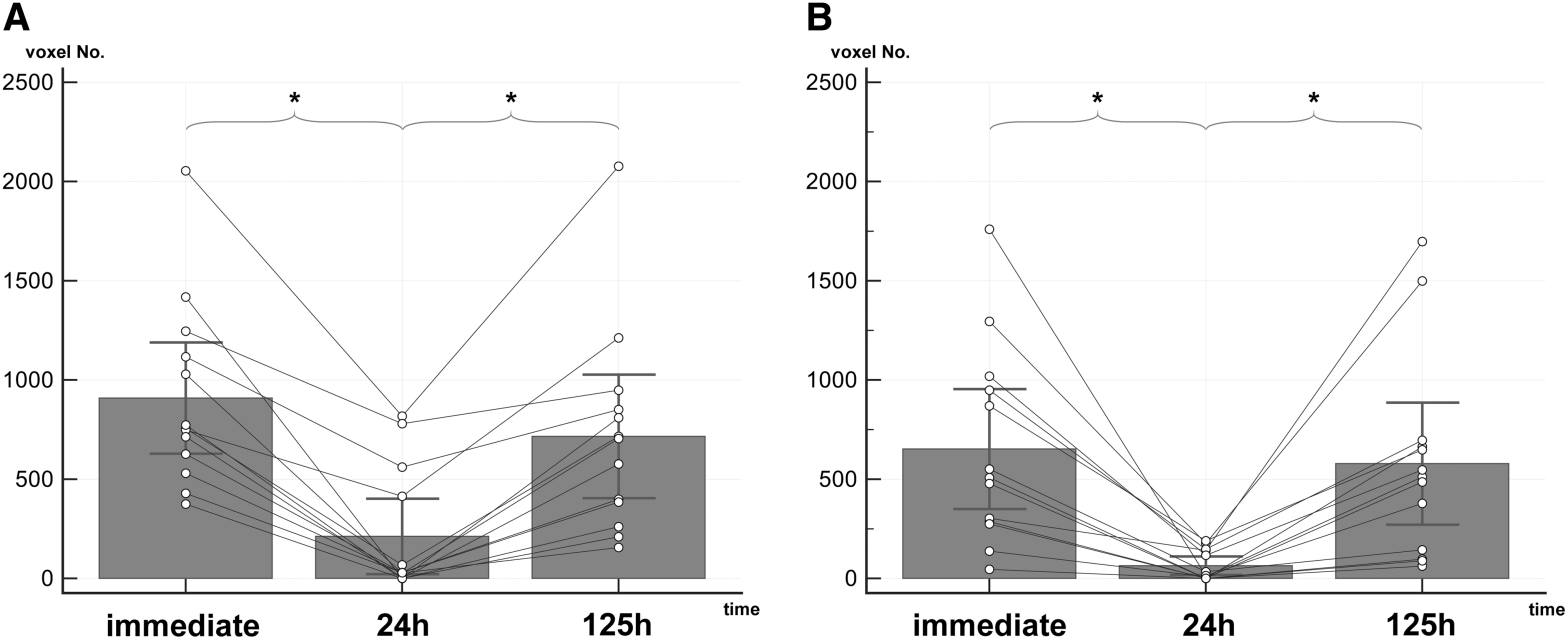
Average temporal susceptibility-weighted imaging hypointensity extent changes at the injury sites in 13 animals. **(A**) Left side (larger gauge injury). **(B)** right side (lesser gauge injury). Asterisks refer to analysis of variance pairwise comparisons between time points resulting in a Bonferroni corrected *p* value of <0.05.

Average hypointensity extents were significantly (corrected *p* < 0.05) smaller at the 24 h time point compared with the immediate and the 125 h acquisitions (Fig. 4A, 4B). Repeated measures ANOVA of the average hypointensity extents of the control parietal bone region did not show any significant (corrected *p* < 0.05) difference among the time points. The results of the ANOVA tests are presented in Table 1.

**Table 1.** Results of Hypointensity Extents Repeated Measures Analysis of Variance

		*Left (larger gauge injury)*	*Right (lesser gauge injury)*	*Control (parietal bone)*
Extent^a^	Immediate	908	652	1958
	24 h	212	64	2134
	125 h	716	578	2286
*p* value^b^	24 h vs. Immediate	< 0.0001	0.0022	1
	24 h vs. 125 h	0.0004	0.0048	1
	Immediate vs. 125 h	0.317	1	0.46

^a^ Number of hypointense voxels in the minimum intensity projection images.

^b^ p values from pairwise comparisons, Bonferroni corrected.

Filtered phase images presented a similar temporal pattern based on visual inspection—i.e., pronounced phase alterations in forms of hyperintensities at lesion sites were present at the immediate and last imaging (125 h) that were less apparent at the 12 h, 24 h, or 48 h time points. Figure 2B shows filtered phase images of the three animals that underwent five imaging time points.

### Histology

**FIG. 5.**
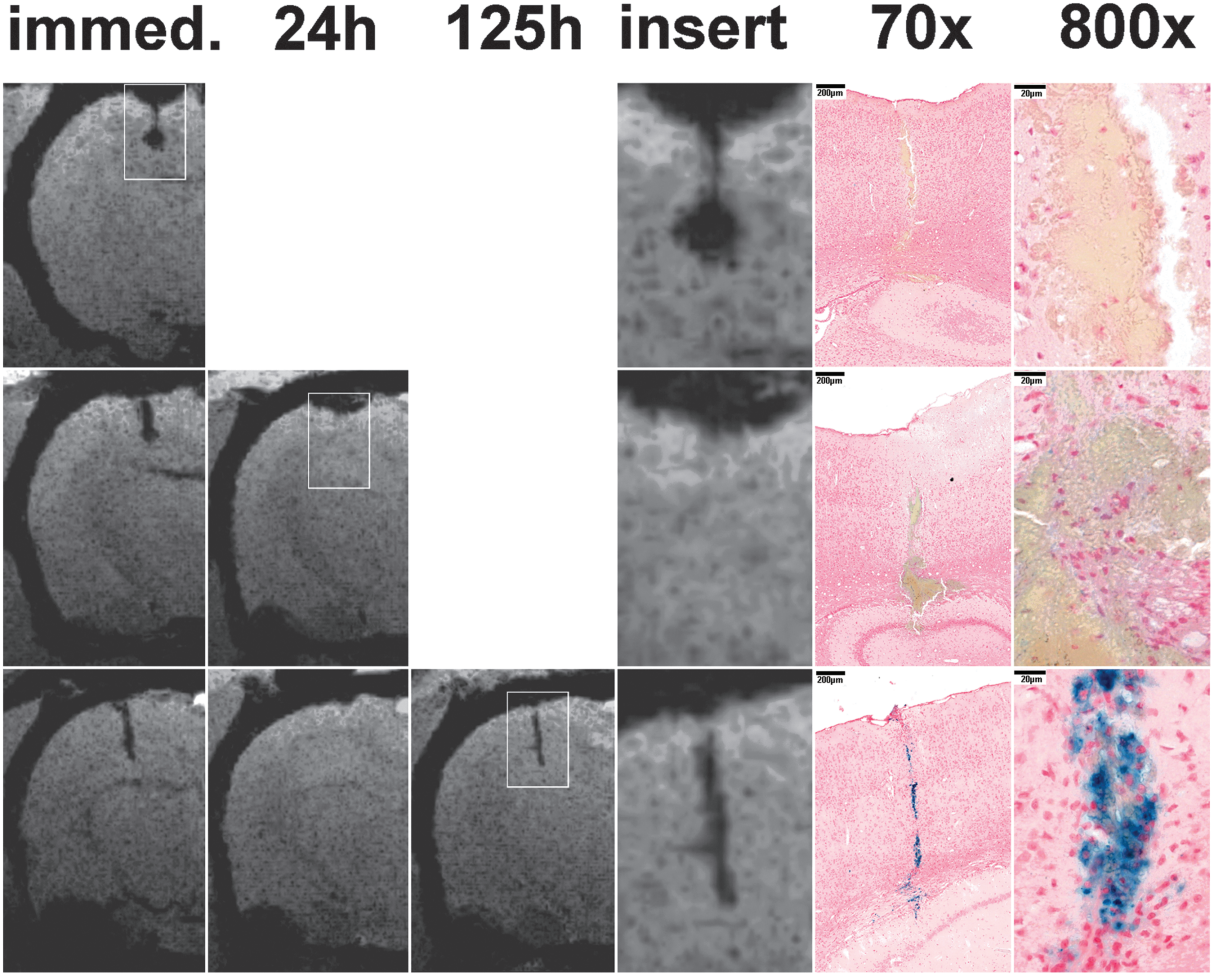
Correlations of susceptibility-weighted imaging (SWI) and histology of microbleeds. First, second, and third lines show rats that were euthanized after the immediate, 24 h, or 125 h imaging, respectively. The available coronal SWI minimum intensity projection images of microbleeds of interest are presented, with inserts indicating the corresponding histological (Prussian blue staining, 70x and 800x) images. Blood products are apparent in the animal euthanized at 24 h and 125 h as well, despite the absence of hypointensities in the 24 h SWIs. Prussian blue staining indicates the presence of hemosiderin at 125 h.

Bleedings were present in all animals at the lesion sites regardless of the time point of euthanasia. In the vast majority of cases, bleedings were macroscopically not visible in the slides, and their diameter (width) was measured to be typically 60–100 μm; at a few sites maximal diameters reached 200 μm. The relatively large circumscribed hypointensities in SWI that were accidentally formed in three animals (second in Fig. 2A, first and ninth in Fig. 3), however, were macroscopically visible in both the paraffin tissue blocks and the slides, measuring 500–900 μm diameters. In animals that were euthanized at 24 h after injury, bleeds were still present as expected based on the immediate imaging, despite the decrease or disappearance of corresponding hypointensities at 24 h imaging. Only by the last (125 h) time point, the presence of hemosiderin was shown by Prussian blue staining. Corresponding representative findings are shown in Figure 5.

## Discussion

We developed an experimental model in which histologically proven brain parenchymal microbleeds were produced and reliably detected by SWI. Microbleeds significantly decreased in extent and often completely became invisible between the immediate and the 24 h acquisition time point. Until the last (125 h) imaging time point, a significant re-increase—i.e., re-appearance—was present. Complete invisibility at 24 h occurred commonly. Histopathological findings confirmed that microbleeds did not actually vanish, because microbleeds were present in all time points of animal euthanasia. These findings highly explain the observations of previous human case reports and an experimental case report on microbleeds appearance changes,^5,30–33^ and at the same time indicate that short-term microbleed change is a general phenomenon, because of MR visibility change over time. In humans, the timing of the temporary disappearance might be somewhat slower. Based on the case observations by Watanabe and coworkers,^32^ microbleed invisibility might take place roughly between 24 h and seven days after formation.

Accidentally, larger blood collections resembling hematomas instead of microbleeds occurred at the injury sites, probably because of an injury of a relatively large vessel. These hematoma-like lesions did not disappear or show obvious changes over time. This implied that the phenomenon of temporary invisibility is dependent on bleeding size.

The explanation of the phenomenon of temporary microbleed invisibility is challenging. Based on the macroscopic hemorrhage literature, a possible reason is the development of methemoglobin that markedly decreases T1 relaxation time because of dipole-dipole interactions.^15,36^ Consequently, T1 shine-through may occur in SWI or T2* GRE images.^18,19,32^ In the present study, however, some arguments can be made against the development of methemoglobin as the explanation for microbleed signal void cessation. First, apparent microbleed signal increase was found as early as 12 h, while methemoglobin is expected to form earliest at 24 h in rats.^18,37^ Second, no T1 shine through or hyperintensity was detected over the lesions. Third, phase effects appeared to be diminished at 24 h, implying the weakening of paramagnetism. That is in contrast with the supposed effect of methemoglobin; methemoglobin is regarded to be just as paramagnetic as deoxyhemoglobin, because of its five unpaired electrons.^15,36,38^

Unlike deoxyhemoglobin, oxyhemoglobin is known to not cause signal void in SWI; therefore, a theoretical explanation for the development of microbleed invisibility might be blood oxygenation. The passive oxygenation of microbleeds in the brain, however, can be excluded, because normal brain tissue O_2_ tension is known to be not higher than venous blood O_2_ tension either in humans or rats.^39,40^ In fact, bleedings of even an arterial source should become and remain deoxygenated.^15^

Another important mechanism possibly affecting MRI appearance in the hyperacute phase is the increase of red blood cell ratio because of clot retraction.^15,22^ An elevation in hematocrit to approximately 90% occurs when extravasated blood settles and subsequently forms a retracted clot.^41^

Some studies presented parabolic correlations between T2 relaxation time and deoxygenated red blood cell concentration, so that shortest T2 times occurred at hematocrits of 50%.^23,42^ It has been postulated that local field inhomogeneity in extracellular water caused by deoxyhemoglobin containing red blood cells results in selective T2 relaxation enhancement (T2 time decrease).^23^ If the blood is concentrated by clotting or settling, the removal of the extracellular water will greatly reduce the effects of T2 proton relaxation enhancement^23,43^; that explains T2 time lengthening at higher hematocrits.

Such hematocrit and local field inhomogeneity dependent T2 relaxation effect is very likely to be linked to the T2* effect as well. Clot inhomogeneity at voxel level is regarded to cause signal loss in GRE.^21,43^ In contrast, full retracted clots despite containing red blood cells with deoxygenized hemoglobin have been shown to increase signal in GRE.^21^ Such samples may paradoxically not cause a marked signal void in SWI either. This is in contrast with the findings of Barnes and associates^44^ who have shown that *in vivo* blood sedimentation because of stasis in veins is related to phase shift and signal decrease in SWI.

In this study, however, the hypointense settled blood fell into macroscopic range and, lacking microscopic analysis, no information could be obtained regarding voxel level inhomogeneity. Therefore, it still can be postulated that homogeneous clot formation caused signal gain at the microscopic level might be the primary reason for the temporary invisibility of microbleeds. Macroscopic hematomas, in turn, are regarded to be composed of partial retracted clots with small plasma pools^20,21,26,45^ maintaining signal void in susceptibility sensitive MRI probably as a result of voxel level inhomogeneity.^21^

The re-appearance of microbleeds from the 24 h to last imaging time points can be explained by the development of late breakdown products of hemoglobin as hemosiderin and ferritin, known to be superparamagnetic.^2,15^

For the MR investigation of microbleeds, this model had some advantages compared with other injury and hemorrhage models, such as the weight drop/fluid percussion trauma models,^46,47^ or autologous blood, collagenase injection,^48^ and lipopolysaccharide^49^ hemorrhage models. Instead of macroscopic hematomas, our model produced true microbleeds, in controlled locations, without unnecessary burdening of the animals—none of the animals were lost or showed any signs of significant morbidity during the examination period.

Still, this study has certain limitations. Because we performed the examination using single hardware and set of parameters, it cannot be excluded that changes in specific parameters would affect the characteristics of this phenomenon. At the same time, the fact that different, 1.5 T and 3 T systems were applied in previous human case reports leading to comparable findings^30,33^ strongly suggests that the phenomenon is not scanner dependent. The mechanism of microbleed formation because of direct vessel injury in the present model is obviously different from the expected more indirect vessel injury mechanisms in traumatic brain injury. The actual circumstances of microbleed formation, however, are very unlikely to substantially influence the later biochemical sequelae of the extravasated blood products.

The phenomenon of short-term temporary microbleed visibility decrease in MRI may have direct clinical and scientific implications. An inadequate timing of MRI for detecting microbleeds may result in false negative findings. In follow-up imaging, microbleed disappearance should not be interpreted as real regression, while appearance should not be misdiagnosed as re-bleeding or hemorrhagic transformation. The latter, however, cannot be certainly excluded when missing hyperacute imaging. The detection of both the disappearance and re-appearance of a microbleed may be the only way to differentiate new microbleeds from chronic ones.

## Conclusion

We developed a reliable experimental model for temporal microbleed MRI examinations and confirmed general temporary reduction in visibility of microbleeds in the acute phase in SWI. Although the exact explanation of this phenomenon remains elusive, our findings prove that imaging timing is important when using SWI acutely as a diagnostic tool for microbleeds.
